# Supplementary material for: Ubiquitylome study identifies increased histone 2A ubiquitylation as an evolutionarily conserved aging biomarker
Source: Nat Commun. 2019 May 21;10:2191. doi: 10.1038/s41467-019-10136-w (PMC6529468; doi:10.1038/s41467-019-10136-w)
Supplement: Supplementary file 1 — Supplementary Information [file 41467_2019_10136_MOESM1_ESM.pdf]

Supplementary Information

Ubiquitylome analysis identifies increased histone 2A ubiquitylation as an evolutionarily conserved aging biomarker

Yang et al.

Supplementary Figure 1-8

Supplementary Table 1

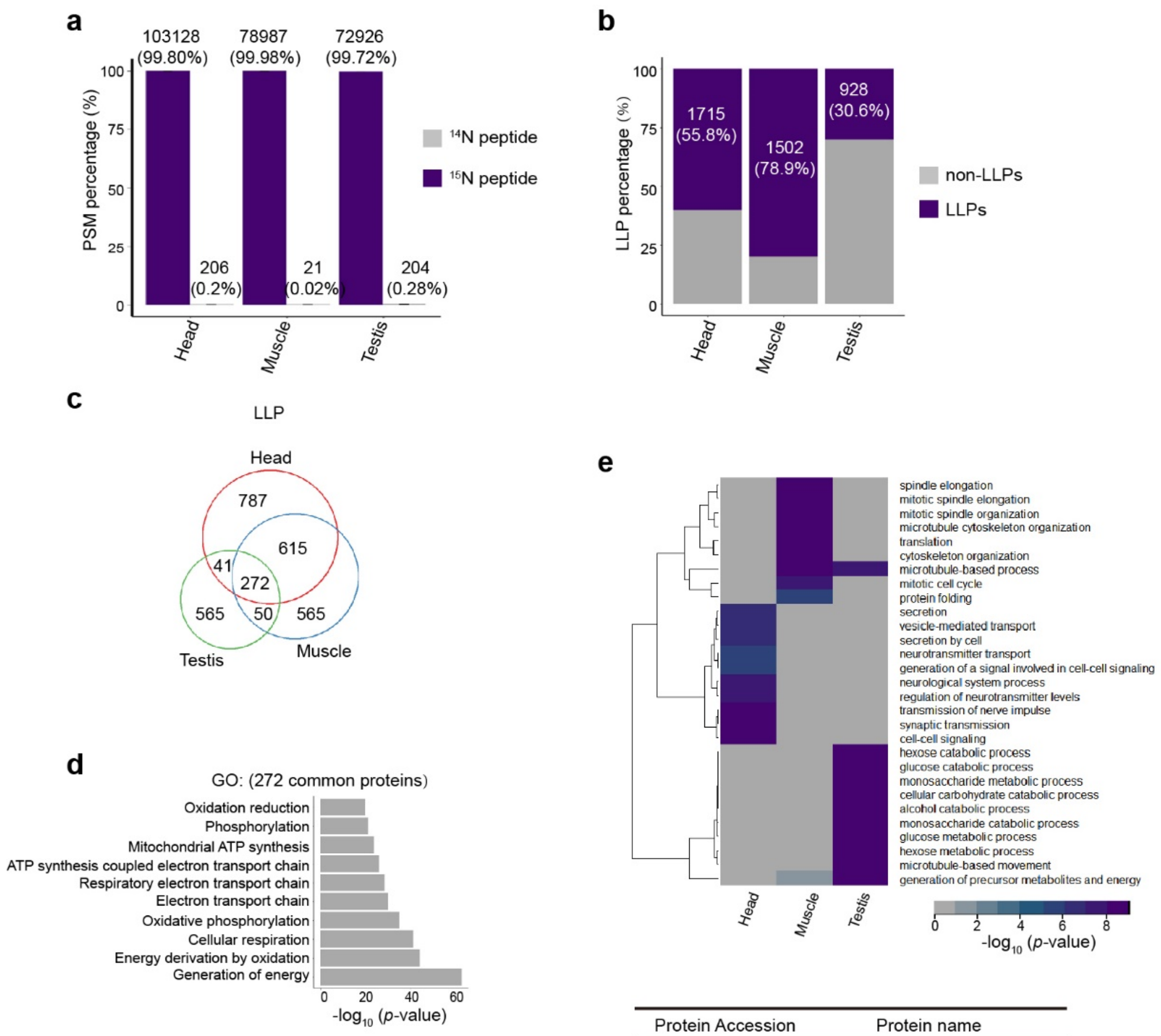

Protein Accession	Protein name
P02299	Histone H3
P84040	Histone H4
A0A0B4KFZ9	Histone H4r
P11046	Laminin subunit beta-1
P15215	Laminin subunit gamma-1
Q00174	Laminin subunit alpha
X2JAW6	Laminin B2
Q9VXZ0	LP22727p
Q9VQM2	NADH dehydrogenase
Q9VM18	Trehalose 6-phosphate phosphatase
Q9W2E8	CG10320
Q9V3L7	GH05682p
Q9V3W2	GM23292p
Q8MSI2	GH15296p
B7Z0B2	uncharacterized
B7Z0B3	uncharacterized

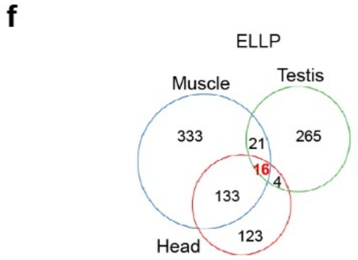

Supplementary Fig. 1 Analysis of long-lived proteome in Drosophila. **a** ^{15}N labeling efficiencies of three tissues. All three tissues were completely labeled with ^{15}N at day 5, as shown by the evidences that over 99% of the total PSMs were identified as ^{15}N forms. **b**

Different tissues have different proportions of long-lived proteins (LLPs). LLP was defined by with more than 10% of ^{15}N form at 60d. **c** Venn diagram of LLPs in three tissues. **d** GO analysis of common LLPs in three tissues. **e** Functional analysis of tissue-specific LLPs. **f** Venn diagram of the ELLPs characterized in Fig. 1c. 16 commonly identified ELLPs in three tissues were listed in the table.

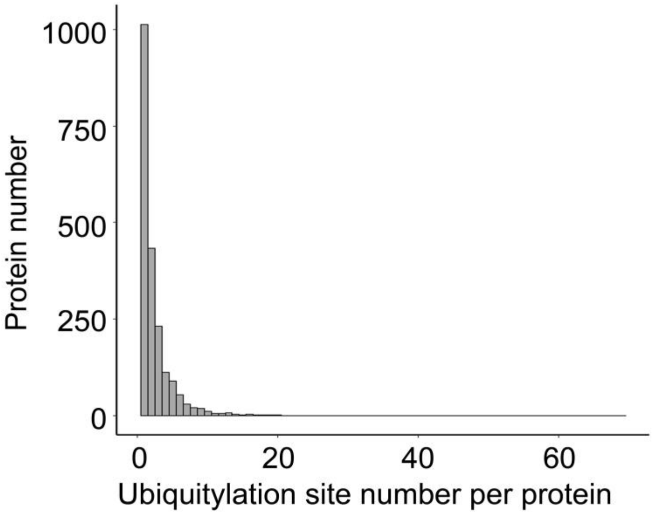

Supplementary Fig. 2 Distribution of the quantified ubiquitylated site number per protein.

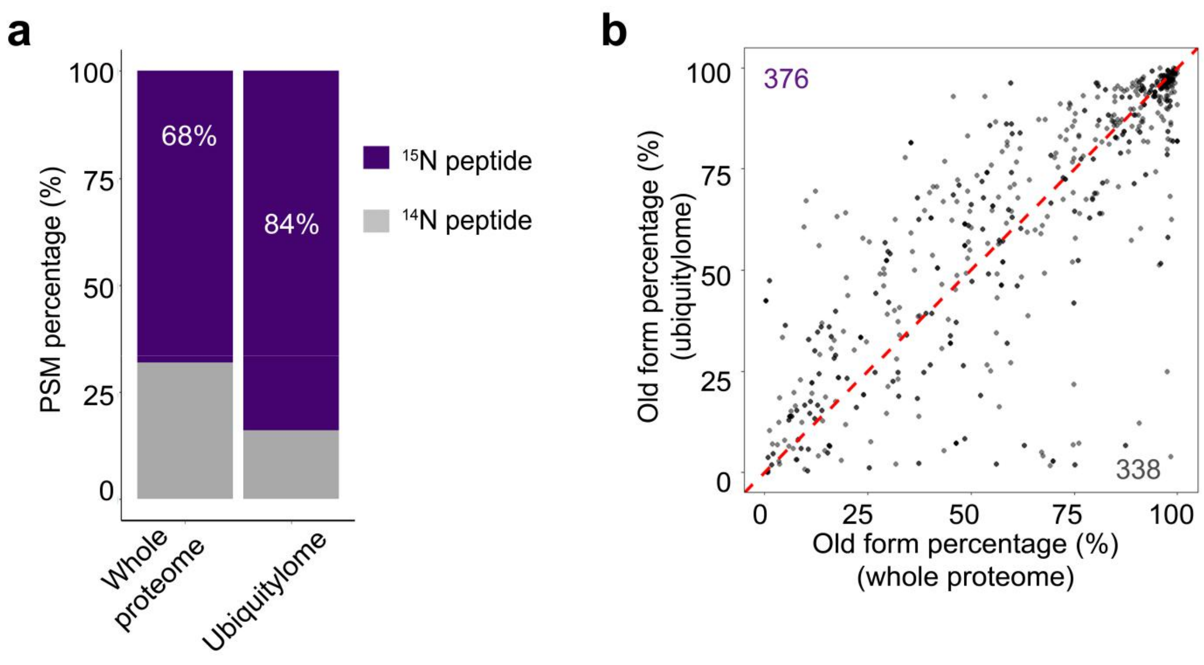

Supplementary Fig. 3 Ubiquitylome analysis of the aging proteome in fly muscle. **a** Proportions of ^{15}N-PSMs in total PSMs. While ^{15}N-labeled old forms accounted for 68% of the entire proteome at 60d, they represented 84% of the ubiquitylated proteome, suggesting increased ubiquitylation in older proteome. **b** Quantifications of ^{15}N signals in total proteins and ubiquitylated proteins.

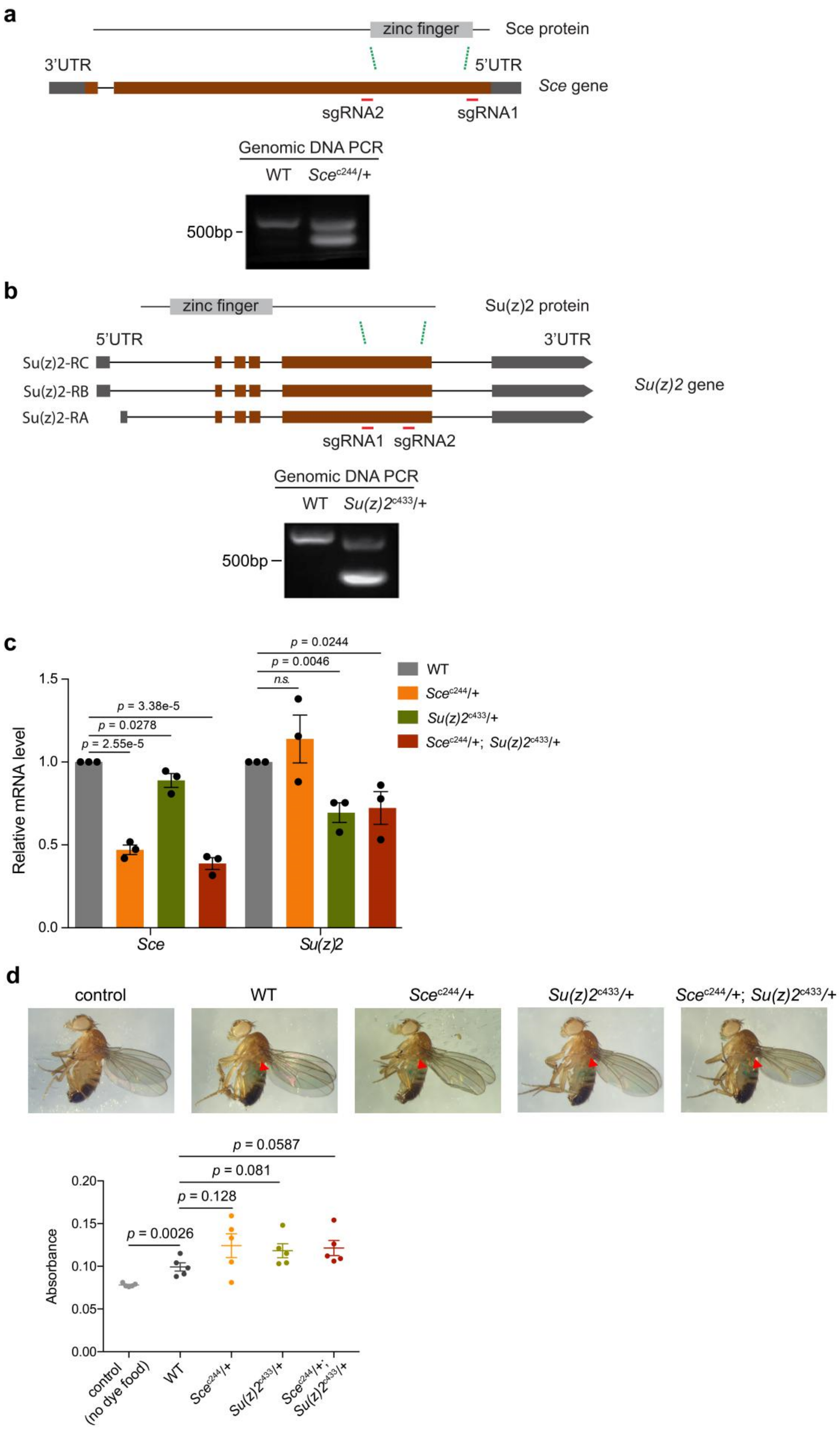

Supplementary Fig. 4 Generation of null alleles for Sce and Su(z)2. **a.b** Map of the *Sce*, *Su(z)2* locus and guiding RNAs used to make *Sce*^{c244}/+ (a) and *Su(z)2*^{c433}/+ (b). To make *Sce*,

Su(z)2 loss of function allele based on the CRISPR/Cas9 method, sgRNA 1 and sgRNA 2, as illustrated in red lines, were used to induce site-specific deletion (top panel). PCR analysis confirms that *Sce*^{c244}/+ and *Su(z)2*^{c433}/+ is a deletion mutant. DNA was from whole flies. Genotypes: control: 5905. *Sce*^{c244}/+. *Su(z)2*^{c433}/+. To name new mutants, a superscript amended to the gene contained a letter c denoting CRISPR/Cas9 method followed by the size of genomic deletion. Resulted mutant flies were backcrossed with 5905 (Flybase ID FBst0005905, w^{1118}) for five consecutive generations to ensure a uniform genetic background. **c** mRNA abundances of *Sce* and *Su(z)* in heterozygous *Sce*^{c244}/+ or *Su(z)2*^{c433}/+ single mutants and *Sce*^{c244}/+; *Su(z)2*^{c433}/+ double mutants (n=3 independent biological repeats for qRT-PCR) (mean±s.e.m.; student *t*-test). qRT-RNA was from heads of 3d old male flies. **d** Fly feeding assay found no significant difference in food intake between WT and mutant flies, as assessed by the appearance of blue dye in the gut (top panel) and the absorbance at 630 nm from whole fly homogenate (bottom panel) (n=5 independent biological repeats for quantification) (mean±s.e.m.; student *t*-test).

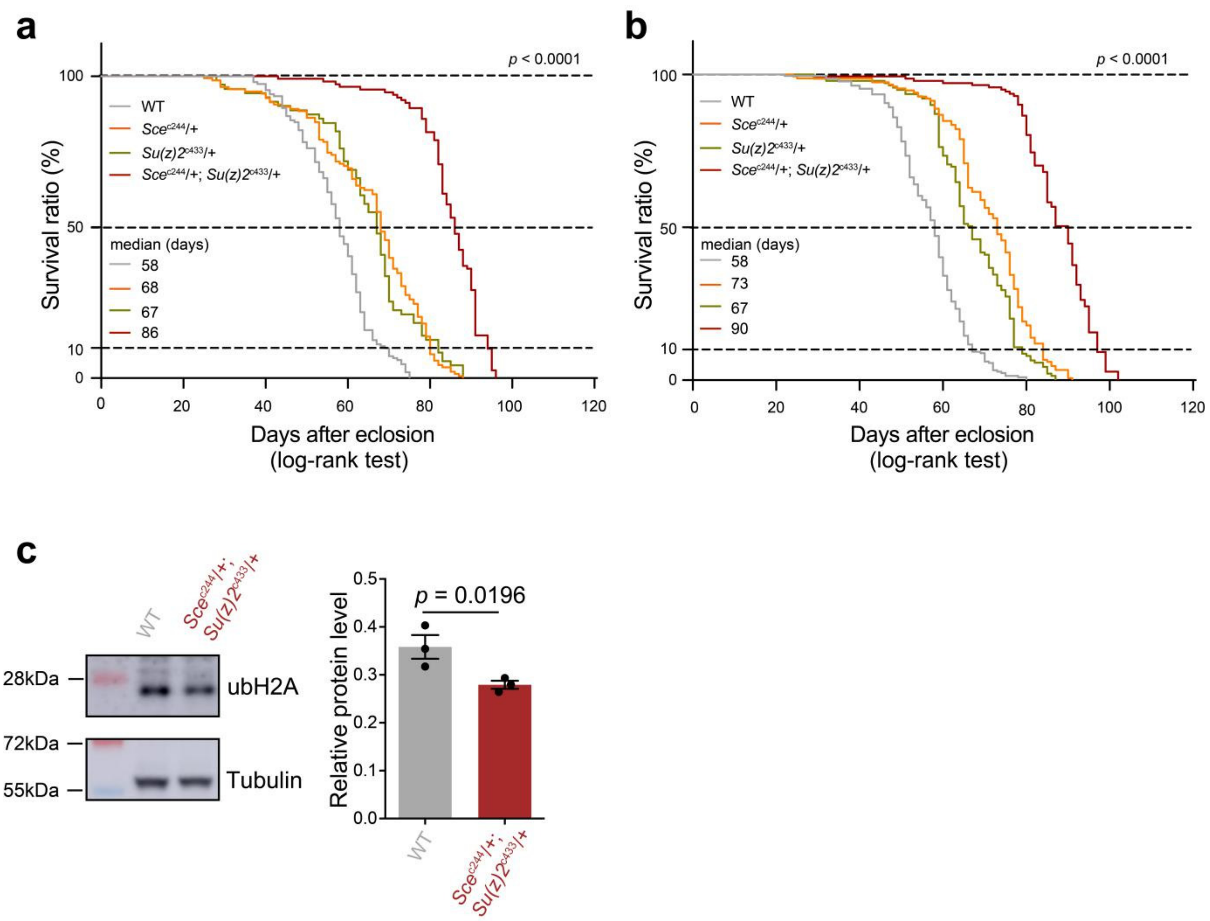

Supplementary Fig. 5 Reducing ubH2A couples with extended longevity in female flies. **a** *Sce*^{c244}/+ or *Su(z)2*^{c433}/+ single mutants and *Sce*^{c244}/+; *Su(z)2*^{c433}/+ double mutants have extended lifespan. Two independent repeats were shown (a,b). (For lifespan: 25 ºC; for each repeat, n>70 female flies per genotype for curve; log-rank test). Genotypes (fly total number (death / censored)): WT: 5905 (151 (151 / 0)). *Sce*^{c244}/+ (138 (138 / 0)). *Su(z)2*^{c433}/+ (71 (71 / 0)). *Sce*^{c244}/+; *Su(z)*2^{c433}/+ (113 (113 / 0)). **b** Independent lifespan assessment for *Sce*^{c244} and *Su(z)2*^{c433} female mutants. Genotypes (fly total number (death / censored)): WT: 5905 (214 (214 / 0)). *Sce*^{c244}/+ (151 (151 / 0)). *Su(z)2*^{c433}/+ (139 (139 / 0)). *Sce*^{c244}/+; *Su(z)*2^{c433}/+ (141 (141 / 0)). **c** ubH2A is significantly decreased in *Sce*^{c244}/+;*Su(z)2*^{c433}/+ flies (n=3 independent biological repeats for ubH2A quantification) (mean±s.e.m.; student *t*-test). Protein was from head tissues of 30d old female heads.

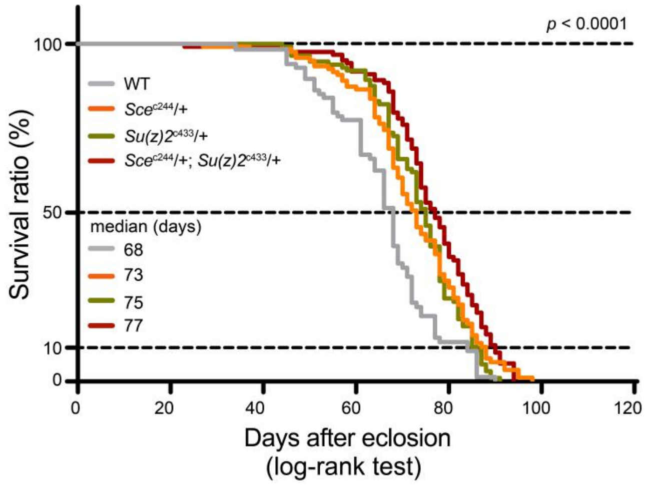

Supplementary Fig. 6 Independent lifespan assessment for *Sce*^{c244} and *Su(z)2*^{c433} mutants. *Sce*^{c244}/+ or *Su(z)2*^{c433}/+ single mutants and *Sce*^{c244}/+; *Su(z)2*^{c433}/+ double mutants have extended lifespan. (For lifespan: 25 ºC; n>100 male flies per genotype for curve; log-rank test). Genotypes (fly total number (death / censored)): WT: 5905 (118 (83 / 35)). *Sc*e^{c244}/+ (122 (102 / 20)). *Su(z)2*^{c433}/+ (115 (102 / 13)). *Sc*e^{c244}/+; *Su(z)*2^{c433}/+ (123 (106 / 17)).

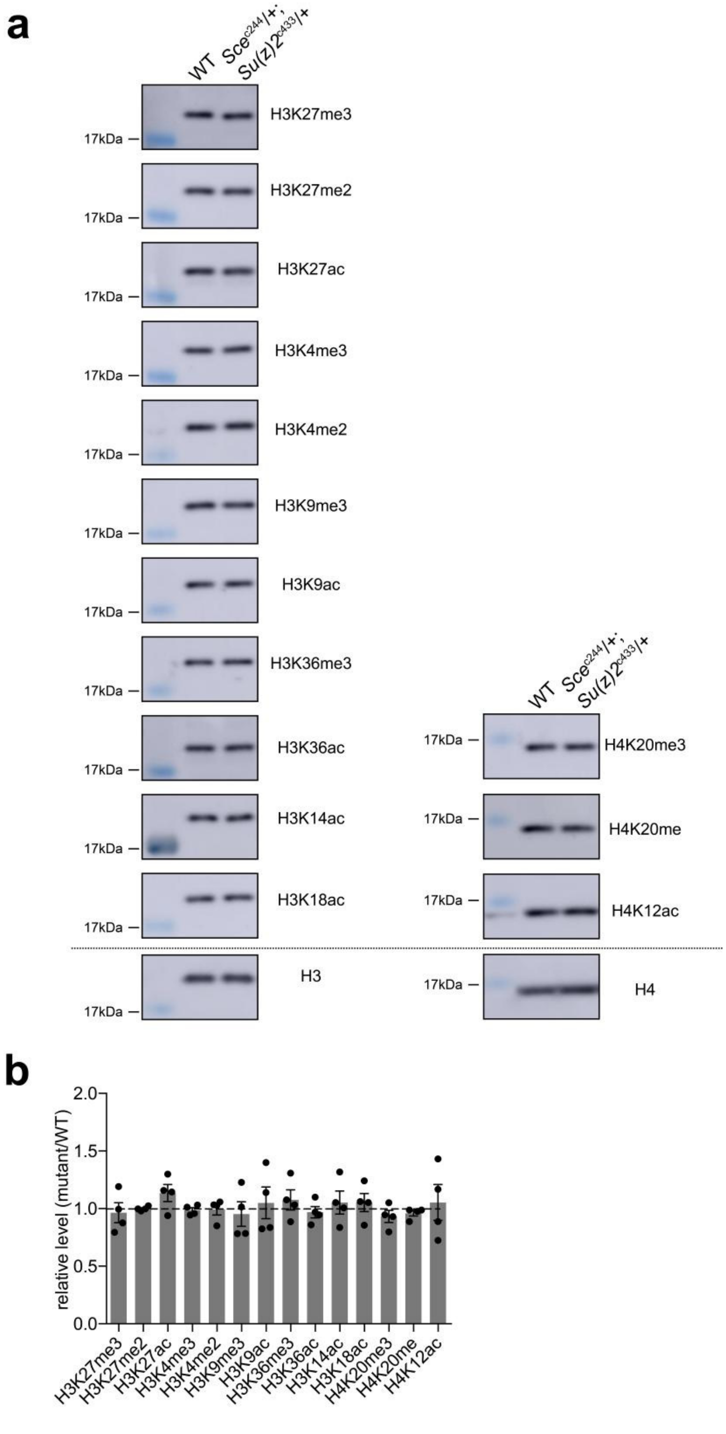

Supplementary Fig. 7 Effect of *Sce*^{c244}/+; *Su(z)2*^{c433}/+ double mutants on selected epigenetic modifications. **a** Western blotting analysis of 14 histone epigenetic markers. **b** Quantification of the western blotting analysis of 14 histone epigenetic markers *Sce*^{c244}/+; *Su(z)2*^{c433}/+ double mutants have a specific reduction on the level of ubH2A, but not other epigenetic markings (n=4 independent biological repeats for western blotting) (mean±s.e.m.; student *t*-test, ** p <0.01). Protein was from heads of 3d old male flies.

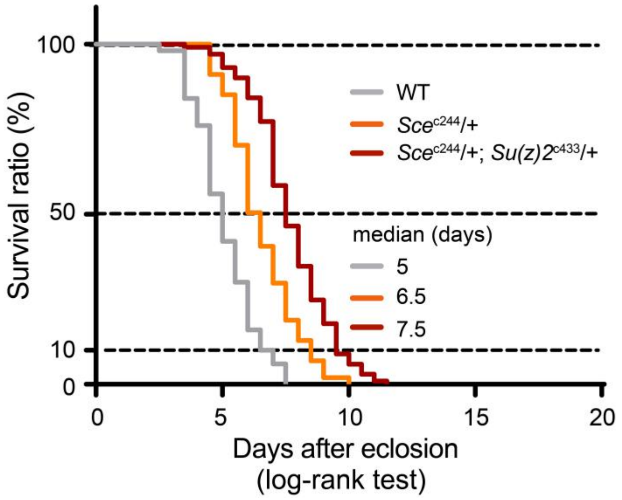

Supplementary Fig. 8 Independent oxidative stress test for *Sce*^{c244}, *Su(z)2*^{c433} double mutants. *Sce*^{c244}/+; *Su(z)2*^{c433}/+ double mutants have enhanced resistance to oxidation than age-matched WT. (for oxidation tests: 25°C, n≥100 male flies per genotype for curve, log-rank test). Genotypes (fly number): WT: 5905 (100). *Sce*^{c244}/+ (101). *Sce*^{c244}/+; *Su(z)2*^{c433}/+ (101). Genotypes (fly total number (death / censored)): WT: 5905 (100 (100 / 0)). *Sc*e^{c244}/+ (101 (101 / 0)). *Sc*e^{c244}/+; *Su(z)*2^{c433}/+ (101 (101 / 0)).

Supplementary Table 1 The ten most abundant ubiquitylated sites on long-lived proteins.

Proteins	Identified sites	#total PSM	Category	Description
E1JHJ4	K1894	42	ELLP	Myosin heavy chain
P13607	K54	41	ELLP	Sodium/potassium-transporting ATPase
A0A0B4LGZ7	K332	37	ELLP	Uncharacterized protein
Q94920	K217	36	LLP	Voltage-dependent anion-selective channel
A1Z7S3	K616	32	LLP	Rab32
Q94920	K52	29	LLP	Voltage-dependent anion-selective channel
P84051	K118	25	ELLP	Histone H2A
Q9VAM6	K83	24	LLP	CDGSH iron-sulfur domain-containing protein
M9PGW0	K351	23	LLP	Innexin
E1JHJ4	K1790	22	LLP	Myosin heavy chain

The abundance of ubiquitylated sites was ranked by the total PSM counts.
