# Supplementary material for: Ubiquitylome study identifies increased histone 2A ubiquitylation as an evolutionarily conserved aging biomarker
Source: Nat Commun. 2019 May 21;10:2191. doi: 10.1038/s41467-019-10136-w (PMC6529468; doi:10.1038/s41467-019-10136-w)
Supplement: Supplementary file 3 — Description of Additional Supplementary Files [file 41467_2019_10136_MOESM3_ESM.docx]

**Description of Supplementary Files**

**File Name:** Supplementary Data 1

**Description:** Quantification of 15N% in head proteins.

**File Name:** Supplementary Data 2

**Description:** Quantification of 15N% in muscle proteins.

**File Name:** Supplementary Data 3

**Description:** Quantification of 15N% in testis proteins.

**File Name:** Supplementary Data 4

**Description:** Quantification of ubiquitylated sites.
