# Supplementary material for: Ubiquitylome study identifies increased histone 2A ubiquitylation as an evolutionarily conserved aging biomarker
Source: Nat Commun. 2019 May 21;10:2191. doi: 10.1038/s41467-019-10136-w (PMC6529468; doi:10.1038/s41467-019-10136-w)
Supplement: Supplementary file 8 — Reporting Summary [file 41467_2019_10136_MOESM8_ESM.pdf]

nature research

Corresponding author(s): Yaoyang Zhang

Last updated by author(s): Apr 2, 2019

Reporting Summary

Nature Research wishes to improve the reproducibility of the work that we publish. This form provides structure for consistency and transparency in reporting. For further information on Nature Research policies, see Authors & Referees and the Editorial Policy Checklist.

Statistics

For all statistical analyses, confirm that the following items are present in the figure legend, table legend, main text, or Methods section.

n/a	Confirmed	
☐	☒	The exact sample size (n) for each experimental group/condition, given as a discrete number and unit of measurement
☐	☒	A statement on whether measurements were taken from distinct samples or whether the same sample was measured repeatedly
☐	☒	The statistical test(s) used AND whether they are one- or two-sided<br>*Only common tests should be described solely by name; describe more complex techniques in the Methods section.*
☒	☐	A description of all covariates tested
☐	☒	A description of any assumptions or corrections, such as tests of normality and adjustment for multiple comparisons
☐	☒	A full description of the statistical parameters including central tendency (e.g. means) or other basic estimates (e.g. regression coefficient) AND variation (e.g. standard deviation) or associated estimates of uncertainty (e.g. confidence intervals)
☐	☒	For null hypothesis testing, the test statistic (e.g. F, t, r) with confidence intervals, effect sizes, degrees of freedom and P value noted<br>*Give P values as exact values whenever suitable.*
☒	☐	For Bayesian analysis, information on the choice of priors and Markov chain Monte Carlo settings
☒	☐	For hierarchical and complex designs, identification of the appropriate level for tests and full reporting of outcomes
☒	☐	Estimates of effect sizes (e.g. Cohen's d, Pearson's r), indicating how they were calculated

Our web collection on statistics for biologists contains articles on many of the points above.

Software and code

Policy information about availability of computer code

Data collection	The MS data were acquired using XCalibur 3.1 from Thermo Fisher Scientific.
Data analysis	The MS data were processed by using software Integrated Proteomics Pipeline - IP2 (Version 4.3.2, Integrated Proteomics Applications, San Diego, CA) and Skyline.

For manuscripts utilizing custom algorithms or software that are central to the research but not yet described in published literature, software must be made available to editors/reviewers. We strongly encourage code deposition in a community repository (e.g. GitHub). See the Nature Research guidelines for submitting code & software for further information.

Data

Policy information about availability of data

All manuscripts must include a data availability statement. This statement should provide the following information, where applicable:

- Accession codes, unique identifiers, or web links for publicly available datasets
- A list of figures that have associated raw data
- A description of any restrictions on data availability

The MS data have been deposited under the accession number PXD013101 at iProX (https://www.iprox.org/page/project.html?id=IPX0001204000) and the Illumina RNA-seq data have been deposited in the Sequence Read Archive (SRA), using the NCBI portal, under the BioProject accession number PRJNA453196 and SRA accession number SRP142472 (https://trace.ncbi.nlm.nih.gov/Traces/sra/?run=SRR7059190).

Field-specific reporting

Please select the one below that is the best fit for your research. If you are not sure, read the appropriate sections before making your selection.

☒ Life sciences ☐ Behavioural & social sciences ☐ Ecological, evolutionary & environmental sciences

For a reference copy of the document with all sections, see nature.com/documents/nr-reporting-summary-flat.pdf

Life sciences study design

All studies must disclose on these points even when the disclosure is negative.

Sample size	The specific animal numbers for western blotting analysis and lifespan analysis were indicated in the method section and figure legends.
Data exclusions	No data was excluded.
Replication	Unless specifically stated, all experiments were done with at least three biological replicates.
Randomization	Sample randomization is not applicable for this study.
Blinding	The investigators were not blinded to group allocation representing different ages during data collection, but blinded in data analysis in this study.

Reporting for specific materials, systems and methods

We require information from authors about some types of materials, experimental systems and methods used in many studies. Here, indicate whether each material, system or method listed is relevant to your study. If you are not sure if a list item applies to your research, read the appropriate section before selecting a response.

Materials & experimental systems

n/a	Involved in the study	
☐	☒	Antibodies
☒	☐	Eukaryotic cell lines
☒	☐	Palaeontology
☐	☒	Animals and other organisms
☒	☐	Human research participants
☒	☐	Clinical data

Methods

n/a	Involved in the study	
☒	☐	ChIP-seq
☒	☐	Flow cytometry
☒	☐	MRI-based neuroimaging

Antibodies

Antibodies used	Primary antibody used for ubH2A detection was diluted 1:2000 (anti-ubH2A, #8240, rabbit host, CST, USA) for Drosophila head, mouse brain and heart, monkey brain, and human brain samples. The following antibodies were used in western blots for fly H3 and H4 epigenetic markers: rabbit anti-H3K27me3 (1:1000, 07-449, millipore), rabbit anti-H3K27me2 (1:1000, ab24684, abcam), rabbit anti-H3K27ac (1:1000, ab4729, abcam), rabbit anti-H3K4me3 (1:1000, 07-473, millipore), rabbit anti-H3K4me2 (1:1000, 07-030, millipore), rabbit anti-H3K9me3 (1:1000, ab8898, abcam), rabbit anti-H3K9ac (1:1000, 06-942, millipore), rabbit anti-H3K36me3 (1:1000, ab9050, abcam), rabbit anti-H3K36ac (1:1000, 07-530, millipore), rabbit anti-H3K14ac (1:1000, 07-353, millipore), rabbit anti-H3K18ac (1:1000, ab1191, abcam), rabbit anti-H4K20me3 (1:1000, ab9053, abcam), rabbit anti-H4K20me (1:1000, ab9051, abcam), rabbit anti-H4K12ac (1:1000, 07-595, millipore), goat anti-H3 (1:1000, ab12079, abcam), and rabbit anti-H4 (1:1000, ab10158, abcam).
Validation	All antibodies used in this research were from commercial sources, and antibodies were validated by manufacturers.

Animals and other organisms

Policy information about studies involving animals; ARRIVE guidelines recommended for reporting animal research

Laboratory animals	The WT fly line used was 5905 (FlyBase ID FBst0005905, w1118). All Sce and Suz(2) mutant fly lines used in this study have been backcrossed with 5905 for five consecutive generations for a uniform genetic background, to assure that phenotypes were not associated with any variation in background.<br>Male C57 mice were used at the ages of 5 months and 23 months, with 4 mice per group.<br>Rhesus macaque parietal lobes were obtained from the brain bank of University of Science and Technology of China. The age and gender of individual animals were indicated in the Figure 3h.

Wild animals	No wild animals were used in this study.
Field-collected samples	No field-collected samples were used in this study.
Ethics oversight	All animal procedures were reviewed and approved by the Institutional Animal Care and Use Committee at Chinese Academy of Sciences and are in accordance with the Guide for the Care and Use of Laboratory Animals of Chinese Academy of Sciences.

Note that full information on the approval of the study protocol must also be provided in the manuscript.
